# A New Anatomically Based Nomenclature for the Roots and Root Canals—Part 1: Maxillary Molars

**DOI:** 10.1155/2012/120565

**Published:** 2011-12-15

**Authors:** Jojo Kottoor, Denzil Valerian Albuquerque, Natanasabapathy Velmurugan

**Affiliations:** ^1^Department of Conservative Dentistry and Endodontics, Mar Baselios Dental College, Kothamangalam, Ernakulam, Kerala 686691, India; ^2^Private Practice, Dental Expert, Ocean View, Bandra West, Mumbai 400050, India; ^3^Department of Conservative Dentistry and Endodontics, Meenakshi Ammal Dental College and Hospital, Alapakkam Main Road, Maduravoyal, Tamil Nadu, Chennai 600 095, India

## Abstract

Numerous terminologies have been employed in the dental literature to describe the roots and root canal systems of maxillary molars. This multiplicity in naming of roots and canals makes the reader susceptible to misinterpretation and confusion. No consensus thus far has been arrived at for defining the names of roots and root canals in maxillary molars, including their various morphological aberrations. The anatomical relation of roots and their root canals were identified and were subsequently named based on definite sets of criteria. A new method for identification and naming of roots and root canal anatomy in maxillary molars, based on their root and canal relationship, was formulated and is presented in this paper. The nomenclature makes certain essential modifications to the traditional approach to accommodate naming of the various aberrations presented in the maxillary molars. A simple, yet extensive, nomenclature system has been proposed that appropriately names the internal and external morphology of maxillary molars.

## 1. Introduction

Nomenclature refers to a set of terms used in communication by persons in the same profession that enables them to better understand one another. The comprehension of these terms aids in diagnosing and treating disease and defects of the teeth [1]. The maxillary molars are one of the most complex teeth by virtue of their multifaceted internal and external anatomy. They are generally described as a group of teeth containing three principle roots namely, the mesiobuccal (MB), distobuccal (DB), and palatal (P) [2]. Variations in its root anatomy have ranged from 1 root to 5 distinct roots [3–6]. The most common canal configuration of maxillary molars is that each root contains a single principle root canal, named according to the respective root in which it is contained as the mesiobuccal, the distobuccal, and the palatal canal. As with the root variations, the root canals of maxillary molars have also presented with a wide range of variations. A single root canal to as many as seven root canals has been reported in maxillary molars [5, 7], with the most commonly reported variation of a second mesiobuccal canal (18–96%) [8, 9]. Furthermore, each root may display abundant root canal configurations highlighting the diversity of the root canal system in maxillary molars.

A literature search revealed that various authors reporting these variations in the maxillary molars have used numerous terminologies to define their roots and canals. The conventional nomenclature for the description of the root canal morphology of maxillary molars has been non-specific, and this ambiguity is even more pronounced with regards to the second mesiobuccal canal which has been variously cited as the MB2, mesiopalatal, second mesiobuccal, and the mesiolingual canal [3, 7, 10, 11]. It has been widely accepted to be termed as “MB2.” Subsequently, the third mesiobuccal canal was termed as “MB3” [12]. Also, various authors have interchangeably used the term “mesiopalatal” to describe both, the MB2 as well as the mesial of the two palatal canals/roots [7, 10, 13]. Additionally, few authors have described the variation of multiple canals within a root by merely mentioning the number of canals (e.g., 2 or 3 palatal canals) [13–15]. Other descriptive terms for naming canals include mesial and distal which rather confound the reader regarding the canal anatomy [3, 16]. Of these various terminologies, the use of numbers to denote additional canals (MB1, MB2, MB3, DB1, DB2, P1, P2, etc.) is very unusual for nomenclature [7, 17–20]. The numbers convey only the presence of an additional canal/s with no descriptive information of the variant canal system.

Table 1 summarizing the several terminologies that have been used in endodontic literature to express the various root and root canal aberrancies, highlights the lack of consistency within the profession in designating similar variations with regards to maxillary molars. Also, a set criterion has not been put forward to clearly define the root and canal anatomy of maxillary molars in its several variations. Additionally, no nomenclature system has been presented to date that simultaneously considers the relationship of the root and the root canal anatomy of maxillary molars. All these factors highlight the need as well as the importance of a nomenclature which would take these factors into consideration for enhanced communication, improved education, and understanding of the variations in the root and its canal systems. The aim of this paper is to propose a new nomenclature to allow for a comprehensive anatomical description of the roots and root canals in maxillary molars.

## 2. Root and Root Canal Nomenclature

### 2.1. Nomenclature for Root Canals

**Figure 1:**
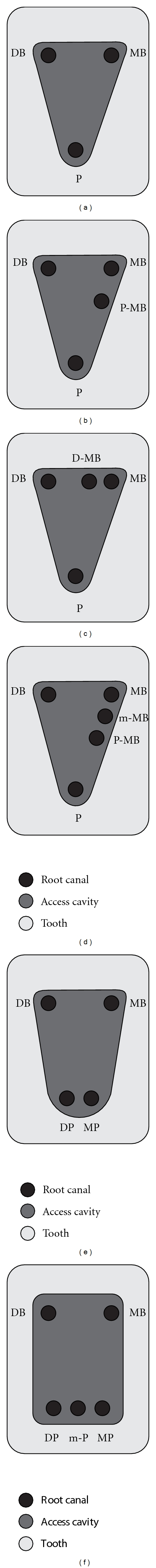
Diagrammatic representation of the various canal configurations in maxillary molars with names according to the proposed nomenclature. (a) The principle canals in maxillary molars are named in accordance with the traditional approach as mesiobuccal (MB), distobuccal (DB), and palatal (P). (b) An additional palatally located mesiobuccal canal is named as palato-mesiobuccal, P-MB. (c) An additional distally located mesiobuccal canal is named as disto-mesiobuccal, D-MB. (d) Two additional palatally located mesiobuccal canals in the mesiobuccal root are named as palato-mesiobuccal, P-MB, and middle-mesiobuccal, m-MB. (e) Two canals in the palatal root are named as mesiopalatal, MP, and distopalatal, DP. (f) Three palatal canals within the same palatal root are named as mesiopalatal (MP), middle-palatal (m-P), and distopalatal (DP).

1. Identification of the principle canals.
  - The principle mesiobuccal or distobuccal canal is that canal whose orifice is located most mesially and buccally or distally and buccally, respectively. For the principle palatal canal, it is the one whose orifice is located most palatally. Also, the path of entrance of the canal can be used to identify the principle canals, whereby the name of the canal is opposite to the path of entrance into the canal. The principle canals would be named as per the traditional nomenclature as mesiobuccal, distobuccal, and palatal canals. These would be denoted by an abbreviation of their anatomical positions as MB, DB, or P for the mesiobuccal, distobuccal, or the palatal canals, respectively, (Figure 1(a)).
2. Additional canal in the MB and/or DB root/s.
  - If a single additional canal is located in proximity to the principle canal and contained in the same principle root, it would be named based on its anatomical position in relation to the principle canal. For naming the additional canal, this anatomical position would be added as a prefix to the name of the principle canal. For instance, a canal-located palatal to the MB canal is named as *palato*-mesiobuccal (*P*-MB) (traditional MB2) (Figure 1(b)). Similarly, a canal located distal to the MB canal is named as* disto*-mesiobuccal (*D*-MB) (Figure 1(c)). This naming is thus self-descriptive of the location of a single additional canal in relation to the principle canal.
  - If two additional canals are located in proximity to the principle canal and in the same bucco-lingual or mesio-distal direction, the location of the canal further most from the principle canal is named based on its anatomical position as a prefix to the principle canal (*palato*-mesiobuccal, *P*-MB). The canal mid-way between the principle canal and the above named canal is named with the prefix *“middle”* (denoted by the letter *“m”),* which is added to the name of the principal canal (*middle-*mesiobuccal, *m-*MB) (Figure 1(d)).
  - The same criteria hold good for the distobuccal canal variations using relevant anatomical names as prefixes to the principle distobuccal canal.
3. For an additional palatal canal.
  - If in case there are two palatal canals in the palatal root, neither of these two canals would be considered as the principle palatal canal. The canals are named based on their mesio-distal location as mesiopalatal (*MP*) or distopalatal (*DP*), thus having no mention of the principle palatal canal (Figure 1(e)).
4. For multiple additional palatal canals.
  - If there are three palatal canals, the central canal is named with the prefix *“middle”* as middle-palatal (*m-P*), while the canals located mesial and distal to this canal would be named as mesiopalatal *(MP)* or distopalatal *(D*P), respectively, (Figure 1(f)).

Thus, as per the proposed nomenclature, a three rooted maxillary molar containing a mesiobuccal canal, an additional palatally located mesiobuccal canal, a distobuccal, and a palatal canal would be denoted as MB, P-MB, DB, and P.

### 2.2. Nomenclature for Roots

**Figure 2:**
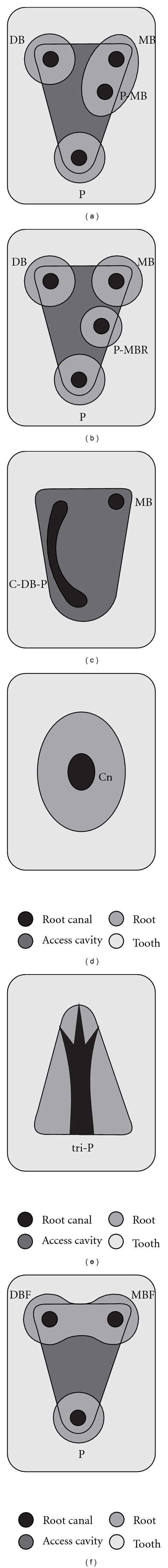
Diagrammatic representations of the various root and canal configurations in maxillary molars named according to the proposed nomenclature. (a) Names of the canals will not be altered if all canals are located in their respective principle roots; MB: mesiobuccal, P-MB: palate-mesiobuccal, DB: distobuccal, and P: palatal. (b) Presence of a palatally located additional root alters the naming of the canals to signify the additional root by use of the suffix *“R.” P-*MBR signifies a *palato*-mesiobuccal canal in a *palato*-mesiobuccal root. (c) Maxillary molar with a C-shaped canal involving the DB and P canals which is denoted as *C-DB-P*. (d) Maxillary molar with a single root and a central canal denoted as *Cn*. (e) Illustration of the palatal root section of a palatal canal with a trifurcation, denoted as *tri-P*. (f) Maxillary molar with fused buccal roots containing distinct MB and DB canal denoted by addition of the letter *F* to the names of the canals in the fused root, that is, MB*F*, and DB*F*.

1. If all canals are located in their respective principle roots, no further modification of the nomenclature is required. Thus, when the canals are named without any mention of the roots, it would signify that the canals are located in their respective principle roots. For instance, a three-rooted maxillary molar with four canals (MB, DB, P, and an additional palatally located MB canal) would be named as MB, P-MB, DB, and P (Figure 2(a)). This would signify that there is no additional root but an additional palatally located canal in the mesiobuccal root.
2. If an additional root is present, the suffix *“R” *should be added, to the name of the canal, based on which principle root it is anatomically associated with. *“R”* should be used as a suffix only to signify the root/s in addition to the principle roots. Thus, in a four-rooted maxillary molar (MB, DB, P and an additional palatally located MB root) with each root having an individual canal, the additional MB root and canal would be named as *P-*MB*R. *Consequently, the root and canal configuration would be MB, *P-*MB*R*, DB, and P (Figure 2(b)).

The proposed formula for naming a root and root canal for maxillary molars according to the present nomenclature is ***XPR***, where *X* is the anatomical position of any additional canal in relation to its respective principle canal (*P*) and* “R”* signifies an additional root.

### 2.3. Modifications for Rare Anatomical Variations

- In cases of C-shaped canals, the prefix *“C”* is added to the canal name. The canal name is expanded to include the extent of the C-shaped canal. For example *C*-DB-P denotes a C-shaped canal configuration which includes both the DB and the P canals. Thus, a maxillary molar containing a distinct MB canal and a C-shaped canal which extends from the DB to the P, the root, and canal configuration of the tooth would be denoted as MB, *C*-DB-P (Figure 2(c)). This naming pattern would also shed light on the possibility of fused roots that contain the C-shaped canal.
- In case of a single-rooted maxillary molar with a single canal, we propose that it can be named as *“Central”* canal, denoted as *“Cn” *(Figure 2(d)). This name more appropriately describes the central location of a single canal within a solitary root.
- Canal variations of a bifurcation or a trifurcation of the main canal at various levels from the orifice have been reported, most commonly in the palatal canal. In such cases, we recommend that the prefix “bifurcation” or “trifurcation”, denoted as* “bi”* and* “tri,” *respectively, be added prior to the name of the canal that is dividing. Thus, a palatal canal that is trifurcating would be named as trifurcation palatal,* tri*-P (Figure 2(e)).
- In cases of fused roots with multiple canals, the canals contained within the fused root would be named based on the previously mentioned criteria for canal nomenclature but with the addition of the suffix *“F,”* instead of the previously mentioned “*R*”. For instance, two canals (MB, DB) within the fused buccal roots with would be named as mesiobuccal-fused and distobuccal-fused; denoted as MB*F *and DB*F*, respectively, (Figure 2(f)).

## 3. Discussion

The use of magnification and newer diagnostic techniques have led to an increase in the number of roots and canals being diagnosed and treated in maxillary molars, thus emphasising the need for an appropriate nomenclature for these canals [7]. When the early studies on the configuration of the mesiobuccal canal were first reported, the newly discovered canal was often referred to as “the second mesiobuccal,” because no one expected more than one canal in this root. However, soon it was called the “mesiolingual” or occasionally the “mesiopalatal” [22]. Terms that have over time gained popularity because of their simplicity, like the use of numbers as in case of the MB2 and MB3, are inappropriate and imprecise names and do not anatomically describe the locations of the canals, having no parallel in endodontic terminology.

**Figure 3:**
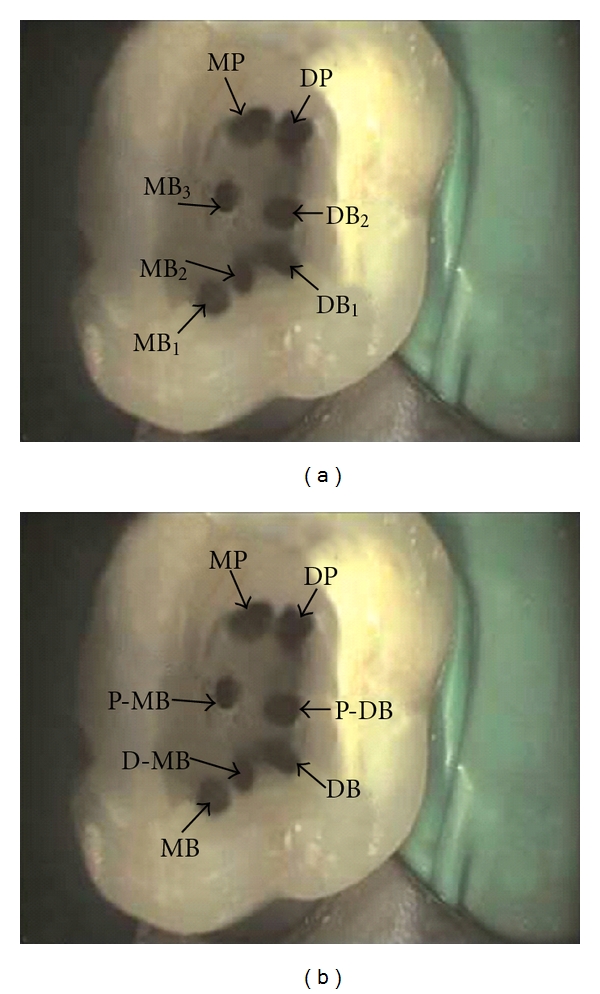
(a) Picture of the access cavity of the maxillary first molar showing the locations of the seven canals contained within the three principle roots and named using the traditional nomenclature. MB: mesiobuccal, DB: distobuccal, DP: distopalatal, and MP: mesiopalatal. (b) Naming of the canals in (a) as per the proposed nomenclature; P-MB: palato-mesiobuccal, D-MB: disto-mesiobuccal, P-DB: palato-distobuccal. (Reprinted with permission from Kottoor et al. [7].)

The “MB2” canal is commonly located palatally and mesially to the “MB1” [8]. However, the additional mesiobuccal canal has been identified at positions other than the conventionally described site. Thus far, it has been the privilege of the author to designate a number that he/she thinks appropriate for that particular eccentrically located canal. For instance, Kottoor et al. described the endodontic management of a three-rooted maxillary molar with seven root canals which were named as MB1, MB2, MB3, DB1, DB2, MP, and DP (Figure 3(a)). The canal located midway between the MB and the DB canals was termed as the “MB2,” while the canal located midway on the line joining the MB and the palatal canals was identified as the “MB3” canal [7]. This underscores the lack of clarity in the traditional approach of naming the canals of the maxillary molar based on their location. As per the proposed classification the root and root canal morphology would be named as MB, *P-*MB, *D-*MB, MP, DP, DB, and *P-*DB (Figure 3(b)), which clearly defines the anatomical positions of these canals.

According to Weine, the name of a canal is opposite to its path of entrance at the level of the canal orifice [22]. However, this does not always hold true and would be imprecise to be used as a rule of thumb to name a canal. For instance, this is not applicable to the so-called MB2 and MB3 canals, as their path of entrance is variable and could be relatively in the same direction. Thus, the naming of a canal only based on its path of entrance at the orifice level is inadequate. Recently, Karthikeyan and Mahalaxmi proposed a new nomenclature for the root canals in maxillary first molars [10]. Although it is a simple modification of the traditional approach and names the additional canals located, the root to canal relationship is not taken into account. For instance, mesiopalatal (MP) and distopalatal (DP) have been proposed as names for the mesiopalatal and distopalatal canals; however information as to whether these canals are contained within the same root or in different roots cannot be inferred. Also, it has not completely done away with the numbering system pointing to the lack of anatomical considerations. Preset names have been specified to canals and any variation other than these cannot be covered under it. For instance, a distally located mesiobuccal canal cannot be named as per their nomenclature.

**Figure 4:**
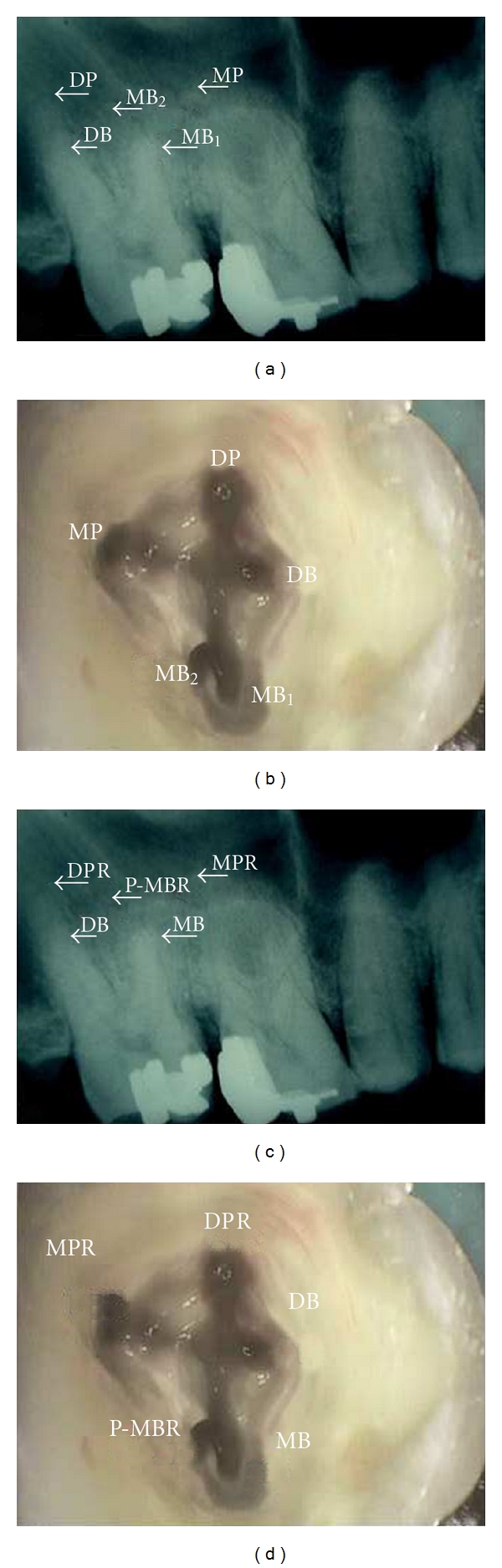
(a) Radiograph showing the root morphology of five separate roots named using the traditional terminology. MB: mesiobuccal, DB: distobuccal, DP: distopalatal, and MP: mesiopalatal. (b) Access opening picture of the same tooth showing the names of the canals as per the traditional approach, wherein each root has a single canal. (c) Radiograph, as seen in (a), with designation of roots according to their anatomical positions as per the proposed nomenclature. “*R*” signifies the presence of an additional root. *P-MBR*: palato-mesiobuccal root, *MPR*: mesiopalatal root, and *DPR*: distopalatal root. (d) Picture, as seen in (b), with naming of canals according to the recommended nomenclature. (Reprinted with permission from Kottoor et al. [4].)

The prognosis of an endodontically treated tooth depends mainly on the adequate cleaning and shaping of the various aberrations of the root and canal system. Thus, giving adequate importance to both, the roots and their canal systems, is imperative for long-term success of endodontic treatment. In addition to the root canal variations, the proposed nomenclature also enables better communication of the root anatomy, especially in cases of additional root(s). For instance, a maxillary second molar with five roots (MB1, MB2, DB, MP, and DP) (Figure 4(a)) and each root containing a single canal were named as MB1, MB2, DB, MP, and DP (Figure 4(b)) [4]. According to the proposed nomenclature, the root and canal configuration would be MB, *P-*MB*R*, DB, MP*R, *and DP*R* (Figures 4(c) and 4(d)). These instances point out to the usefulness of the proposed classification in giving a clear picture of the existing root and canal aberrancies in the maxillary molar.

 The salient features of the proposed nomenclature are that it is based on the anatomical locations of roots and canals, describes the root to canal relationship, is elaborate to cover various aberrations of the root and root canal anatomy; yet is simple, self-explanatory, easy to understand, and communicate. A certain paradigm shift has been adopted for the proposed nomenclature, but a genuine effort has been made to use the traditional naming system whenever it permitted for an accurate anatomical description of roots and their canals. This would augment superior acceptance among fellow clinicians' and researchers alike while simultaneously avoiding any possible confusion arising from usage of an entirely separate set of terms. The proposed nomenclature has taken into consideration previously reported root and canal variations in maxillary molars. Given the nature of unpredictability in the endodontic field, certain aberrations could be reported in the future that may not have been covered under the ambit of the present nomenclature. However, it is the view of the authors that minor modifications in the form of additional criteria would enable their inclusion within the proposed nomenclature.

## 4. Conclusion

The proposed anatomically based nomenclature is simple and self-explanatory, which takes into account a holistic view of the root to root canal relationship. It also defines appropriate terminologies for the numerous anatomical variations that have been previously reported in maxillary molars.

## Figures and Tables

**Table 1:** Variations of roots and the canal anatomy of maxillary molars, as reported by various authors, with the numerous terms that have been used to name these aberrancies.

Root nomenclature	Root canal nomenclature	Reference
MB, DB, ***MP***, DP	MB, DB, ***MP***, ***DP***	Di Fiore, 1999 [20]
MB, ***D***, P	*MB1, MB2*, *P1, P2*, ***D***	Johal, 2001 [16]
MB, DB, P	*MB1, MB2*, DB, ***P1, P2, P3***	Maggiore et al., 2002 [17]
MB, DB, P	*MB1, **MB2, MP***, DB, P	Favieri et al., 2006 [11]
MB, DB, P	*MB1, MB2*, DB***, P1, P2***	Aggarwal et al., 2009 [18]
MB, ***MP***, P, DB	MB, ***MP***, ***M***, P, ***DP***, DB	Adanir, 2007 [3]
MB, DB, P	MB, MP, DB, ***3P***	Pasternak Júnior et al., 2007 [13]
MB, DB, **1st** **P, 2nd* P ***	MB, DB, ***P1, P2***	Ulusoy and Görgül, 2007 [19]
MB, DB, P	MB, DB, ***2P***	Poorni et al., 2008 [15]
MB, DB, P	***MB1, MB2***, ***MB3***, DB, P	Ozcan et al., 2009 [12]
MB, DB, P	***2MB, 2DB, 2P***	de Almeida-Gomes et al., 2009 [14]
***MB1, MB2***, DB, ***MP***, DP	*MB1*, ***MB2***, DB, ***MP, DP***	Kottoor et al., 2010 [4]
MB, DB, P	*MB1, MB2*, ***MB3***, ***DB1, DB2***, ***MP, DP***	Kottoor et al., 2010 [7]
MB, DB, P	*MB1, MB2*, ***DB1, DB2***, ***MP, DP***	Albuquerque et al., 2010 [21]
MB, DB, P	*MB, **SMB, SDB, DBP, MBP**, *	Karthikeyan and Mahalaxmi, 2010 [10]
	*DB, **MP, DP***	

MB: mesiobuccal, DB: distobuccal, P: Palatal, MP: mesiopalatal, DP: distopalatal, M: mesial, D: distal, SMB: second mesiobuccal, MBP: mesiobuccopalatal, SDB: second distobuccal, DBP: distobuccopalatal.
